# Primary health care nurses’ knowledge practice and client teaching of early detection measures of breast cancer in Ibadan

**DOI:** 10.1186/1472-6955-11-22

**Published:** 2012-10-29

**Authors:** OAbimbola Oluwatosin

**Affiliations:** 1 Department of Nursing, Faculty of Clinical Sciences, College of Medicine, University of Ibadan, Ibadan, Oyo State, Nigeria

**Keywords:** Primary health care nurses, Breast self-examination, Clinical breast examination, Mammography, Client teaching

## Abstract

**Background:**

Early detection of breast cancer is vital to effective management and outcome of breast cancer. It has been suggested that women given information and instruction about breast self- examination and breast awareness by health care professionals demonstrated higher knowledge and confidence and tend to practice breast self-examination more than those who received information from other sources. Breast Self-Examination (BSE) and Clinical Breast Examination (CBE) have been recommended as Early Detection Measures (EDM) for developing countries. This study evaluated Primary Health Care (PHC) nurses’ knowledge, practice and client teaching of EDM of breast cancer.

**Methods:**

A descriptive study that utilized stratified random method to select PHC settings for the study. Data was collected from 120 trained nurses in selected settings. This represented 66.3% of total population of PHC nurses (181) in Ibadan. The instrument for data collection was a structured questionnaire that explored the bio data of participants, knowledge, practice and client teaching of EDMs of breast cancer. Ethical approval was obtained from the Ethical Review Committee of Oyo State Ministry of Health, Nigeria.

**Results:**

The mean age of the participants was 44.4±7.5 years. About half (52.2%) were double qualified (Registered Nurse and Midwife). Only 23 (20.0%) of the participants considered painless lump as an early sign of breast cancer while 47 (40.9%) considered pain as an early sign. BSE was listed as EDM of breast cancer by 80.9% of the participants while 40% and 30% listed CBE and mammogram respectively. Only eight (7.9%) have had a mammogram. The logistic regression of client teaching on four variables showed that for every increase in knowledge of breast cancer the odds of client teaching significantly increased by 7.5% (95% CI = 1.27 - 1.125). There were also significant relationships between knowledge of EDM, practice of BSE and client teaching.

**Conclusions:**

It is vital that attention should be given to enhance breast cancer EDMs among the PHC nurses to be able to enhance health deviation self-care of the clients. Nurses’ knowledge, practice as well as client teaching especially at PHC level, will contribute to early detection of breast cancer.

## Background

It is the consensus in the literature that breast cancer is the leading cause of death from cancer among women, accounting for 16% of cancer deaths [1,2]. The picture is not different in Nigeria where breast cancer is the leading cancer in most regions [3,4]. The burden of cancer is very high in developing countries as 62% of the 7.6 million cancer deaths worldwide are from developing countries [5]. A 75% increase in cancer incidence has also been projected in these countries between 2000 and 2020. With advanced technology for early detection and screening, clients have better chance of cure if they utilize these measures [1,6]. Lack of awareness of early detection measures and screening for breast cancer is common in developing countries [7]. Modest or limited health care workers’ training in basic principles of oncology and sometimes even a lack of awareness of the curability of cancer in developing countries has been reported [7]. Furthermore advances in technology that have improved the process of early detection as well as early diagnosis and treatment are either not available or accessible to the public in the developing countries. In view of this and in line with the international breast cancer experts’ recommendations and reported effective experiences [8,9] clinical breast examination (CBE) as the main screening method has been proposed [10,11].

The youngest age of incidence of breast cancer in Nigeria has been reported to be 16 years while the peak age of incidence was 42.6 years [3,4]. Twelve percent (12%) of cases occurred before 30 years while postmenopausal women accounted for 20% of cases [3,4]. This implies that women within the child bearing age who are already at risk of morbidity and mortality from reproductive issues have additional burden.

Research reports have shown that women given information and instruction about BSE and breast awareness by health care professionals demonstrate higher knowledge and tend to practice BSE more than those who received information from other sources [12]. Nurses can utilize specially designed educational programmes in different settings to reach out to the public with vital information about breast cancer early detection measures. Community outreach strategies that are guided by the social and cultural setting will be effective. Since nurses have the tendency to have major influence on women’s behaviour it is very important that they are very knowledgeable about breast cancer and its early detection measures [13]. Nurses are seen as a vital component of the health care team since the nature of their everyday work provides opportunity to encourage and influence women to be breast aware as well as ensure the success of breast cancer screening program [14].

In Nigeria late presentation of breast cancer has been consistently reported for over three decades [15]. Research reports have also shown that women lack knowledge of early detection measures [15,16]. However among health care workers most studies reported high knowledge of breast cancer and it’s Early Detection Measures (EDM) but poor practice of EDM [17,18].

The fact of persistent late presentation of breast cancer for over three decades in an environment where there is absence of an established national screening program for breast cancer and low level of awareness of EDMs is a public health concern. This calls for an exploration of nurses’ knowledge, practice and client teaching of EDM of breast cancer. Most studies both in developed and developing nations have explored and reported health care workers knowledge and practice of early detection of breast cancer and its EDMs but there is limited report of client teaching on various aspects of breast cancer especially in Nigeria. This study explored client teaching and performance of clinical breast examination, a midway approach to the issue of EDM of breast cancer in developing countries.

## Methods

This is a descriptive cross sectional study that utilized stratified random sampling method to select five rural and three urban local government areas from the eleven local government’s areas in Ibadan, Oyo State of Nigeria. This was done to ensure good coverage and a sample population that will be representative of nurses in the local government areas.

Purposive total sampling was used for selection of participants. All the trained nurses in the selected Primary health care centers constituted the sample size of 120. This number represents approximately 66.3% of total population of nurses (181) in the eleven local government areas in Ibadan.

The instrument for data collection was a structured questionnaire developed by the author. The questionnaire has three sections with a total of 54 items. Section A explored participants’ socio-demographic characteristics. Section B assessed knowledge of breast cancer and its early detection measures and section C evaluated practice and client teaching of early detection measures of breast cancer.

Data collection: In each of the Primary health care center selected for the study the nurses were contacted through the primary health care coordinator and or the most senior nurse in the center. A meeting of all nurses was called and the purpose of study was discussed. Questionnaires were distributed to consenting eligible participants. Questionnaires were collected back on the appointment given usually within a week. In all the centers all the trained nurses were eligible as there had been no new employment immediately prior to the study.

Data analysis was by means of descriptive and inferential statistics using the Statistical Package of Social Sciences (SPSS version 16). Knowledge score was calculated by assigning a score of 1 to correct response, 0 to wrong response and no score was assigned to “I don’t know”. Knowledge score of risk factors, early warning signs and treatment of breast cancer were pulled together as knowledge of breast cancer. The maximum expected score for breast cancer was 26 and minimum was 0. Using the same scoring system knowledge of early detection measures of breast cancer was determined. The expected maximum score was 18 and minimum score was 0. The scores for knowledge of breast cancer and knowledge of early detection measures were pulled together as Grand cumulative score of breast cancer.

Score for practice of BSE was calculated by assigning a score of 2 to “always” 1 to occasionally and 0 to “never”. The maximum score obtainable was 36 and minimum was 0. Client teaching was scored after the pattern of practice score. The maximum score obtainable was 12 and minimum was 0. The scores were calculated in percentages and then categorized into four levels: 0-30%, 31-50%, 51-70% and >70%.

Descriptive statistical analysis using crosstabs was done to test for significant association between socio demographic characteristics of age and professional education and Knowledge of breast cancer and EDMs. Regression was performed using percent scores. Knowledge score of breast cancer and knowledge score of early detection measures of breast cancer as independent variables was regressed against practice score of BSE as dependent variable.

The client teaching score was categorized into two categories because the scores did not follow a normal distribution as 41(35.7%) of the population had a score of zero. Hence the two categories were those that scored 0 and those that scored from one and above. Logistic regression of client teaching was done on the following variables: knowledge score of breast cancer, knowledge score of EDM, practice score of BSE and professional qualification which was also a categorical variable group of participants with RN, RM, RN/RM and those with these plus Public health and Community Health officers certificate.

### Inclusion criteria

Only registered nurses who had been working in PHC setting for a minimum of three months were eligible to participate in the study.

### Ethical approval

Ethical approval was obtained from Ethical Review Committee of Oyo state Ministry of Health. Written consent was also taken from the participants after due explanation of study.

## Results

The response rate was 95.8% as 115 out of 120 questionnaires were adequate for analysis. The age of participants ranged between 23 and 59 years. The mean age of participants was 44.4 ±7.5 years. All the respondents were female. Table 1 shows details of socio demographic data of respondents.

**Table 1:** Socio demographic characteristics of respondents

	**Frequency**	**%**
	**N=(115)**	
**Gender**		
**Female**	115	100
**Male**	nil	
**Marital Status**		
**Married**	103	89.6
**Separated**	1	0.9
**Widow**	1	0.9
**Single**	6	5.2
**Age group**		
**23**-**30**	7	6.1
**31**-**40**	22	19.1
**41**-**50**	55	47.8
**51**-**59**	16	13.9
**Educational Qualification**		
**Diploma Certificate**(**RN**,**RM**, **PHN**,**CHO**)	101	87.8
**BSc**/ **B**.**N**.**Sc**	5	5.2
**Non Nursing Bachelor**’**s degree**	1	0.9
**Professional Qualification**		
**Registered Nurse **(**RN**)	1	0.9
**Registered Midwife **(**RM**)	3	2.7
**RN **&**RM**	60	52.2
**RN**,**RM **&**Public Health Nursing **(**PHN**)	11	9.6
**RN**,**RM**,**PHN**&CHO*	37	32.2
**Work Experience **(**Post RN**)		
**4**-**10years**	2	1.7
**11**-**20**	10	8.7
**21**-**30**	12	10.4
**31**-**34**	3	2.6
**Work Experience at PHC facility**		
**1**-**10**	15	13.0
**11**-**20**	6	5.2
**21**-**30**	9	7.8

*CHO Community Health Officer’s certificate.

### Knowledge of breast cancer

Only four (3.5%) of the participants correctly acknowledged that breast cancer is the leading cause of cancer death while two others considered it as the second. Eighty (69.6%) participants acknowledged breast lump as an Early Warning Sign (EWS) of breast cancer. Twenty three (20%) considered that it is a painless lump another four described the lump as hard with irregular border. The participants also mentioned other signs that they considered as early warning signs of breast cancer. Three (2.6%) indicated abnormal lactation while 43 (37.4%) considered bloody nipple discharge. Seven (6.1) considered retraction of the nipple while 47 (40.9%) considered pain as an early warning sign of breast cancer.

Eighty one (70.4) correctly indicated the three forms of treatment of breast cancer as chemotherapy, surgery and radiotherapy. The mean score of knowledge of cause/risk factor was 5.3 ± 1.7, for early warning signs of breast cancer 5.6±1.9, and for knowledge of treatment 6.6±1.9. For the overall knowledge of breast cancer mean score was 17.5±3.5. Socio-demographic characteristics of age and professional qualification were not significantly associated with knowledge of breast cancer, p > 0.05.

### Knowledge of early detection measures of breast cancer

**Figure 1:**
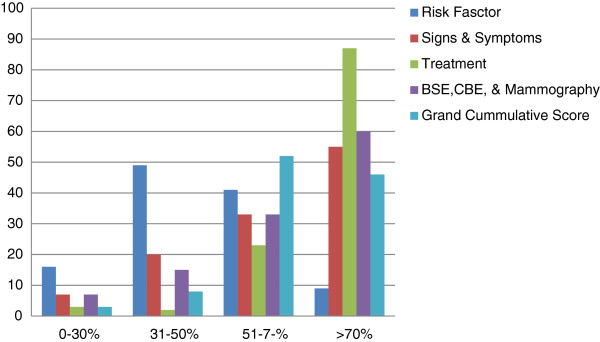
Respondents’ Knowledge Score in various aspects of breast cancer and early detection measures.

The most acknowledged EDM of breast cancer was BSE, by 93 (80.9%) while 40% and 30% acknowledged CBE and mammogram respectively. Seventy one (61.7%) knew that BSE is performed monthly and only 47 (40.8%) knew the correct timing which is 2–3 days after menstruation or a specific day of the month for post menopausal women. For frequency of CBE, 46 (40%) indicated twice in a year while nine (7.8%) suggested once a year; another nine also suggested once every month hence 12 times in a year. In response to the question whether CBE should be performed at every visit to the health facility, 65 (56.5%) indicated “yes”, 29 (25.2%) indicated “no”, while 11 (9.6) indicated they did not know. Ninety nine (86%) considered that the advantage of regular BSE is early detection and prompt treatment. However, 33 (28.7%) indicated death as the danger of not conducting BSE regularly; 10 (8.7%) identified mastectomy and 65 (56.5%) indicated developing cancer as the danger. In response to the question that asked participants to list early detection measures of breast cancer: thirty five (30.4%) indicated mammography, 13 (11.3%) indicated biopsy, 13 (11.3%) indicated diagnostic radiology, 4 (3.5%) chemoprevention, 11 (9.6%) lumpectomy, while 6 (5.2%) medical checkup and another two (1.7%) indicated proper history taking. The mean score for knowledge of EDM was 11.8± 3.2. Figure 1 shows the scores of knowledge of various aspects of breast cancer and its EDM.

### Practice of early detection measures of breast cancer

#### Personal practice

Fifty four (47%) of the participants indicated that they performed BSE monthly, but only 25 (21.7%) performed BSE monthly in the last 12 months. Eight participants (7.0%) had had a mammogram done; seven of them were within the age range of 41–60 while one was in the age range of 23–30. Three indicated that they had it at the University College Hospital, Ibadan.

#### Facility practice

Ninety six (83.5%) indicated that there was no organized program for early detection of breast cancer in their health facility. Eleven of the participants affirmed that there was organized EDM program for breast cancer in their facility. Further analysis showed this was an occasional event, three of them indicated that it was done once, two said it was done during a “doctors week celebration” and one indicated “when we had a seminar” the other five did not specify. Eight (7.0%) indicated that only doctors, 13 (11.3%) indicated that only nurses while 12 others indicated that both nurses and doctors conduct CBE in their facility. Fifty two (45.2%) of the study population had ever conducted CBE but 45 (86.5%) of these were confident of their skill to perform CBE. Thirty seven (71.2%) claimed to have discovered a lump during conduct of CBE for clients. Twenty six referred the clients to secondary or tertiary institution, 10 referred to the doctor in their facility while one indicated that she observed for one month after which the lump disappeared. Eighty six (74.8%) participants were of the opinion that CBE should be conducted at every visit to a health facility to increase early detection of breast cancer.

### Client teaching of EDM of breast cancer

**Figure 2:**
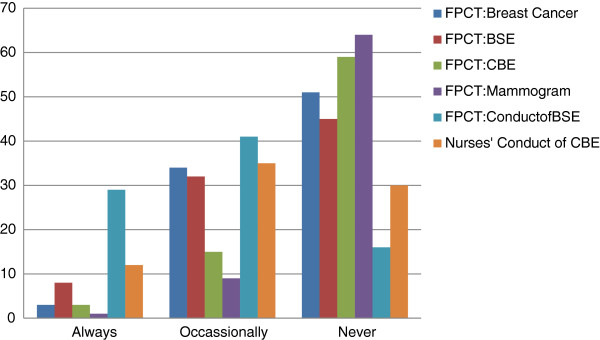
**Client teaching of EDM of breast cancer. **Key: FPCT= Formal Planned Client Teaching.

Only three (2.6%) of the participants indicated that there was Formal Planned Client Teaching (FPCT) for breast cancer in their facility. Teaching clients how to perform BSE was the most practiced and only 29 (25.2%) indicated doing it always. Figure 2 shows details of FPCT as well as conduct of CBE.

Sixty six (57.4%) were taught BSE in formal schools either School of Nursing, Midwifery or of Family Planning. For CBE, 37 (32.2%) were taught either in School of Nursing or Midwifery. Five (4.3%) and six (5.2%) had training about BSE and CBE respectively at seminar/workshop. Fifty eight (50.4%) were aware that mammography was now available at the tertiary institution, the University College Hospital but only six (5.2%) ever informed clients of this development. Several reasons emerged for not giving information to clients about availability of mammography at the University College Hospital. Thirty one (39.9%) acknowledged that they did not have any information on mammography, seven (6.1%) indicated that clients did not come for counseling, and nine (7.8) that they did not have any training about mammography while another four (3.5%) attributed their not informing clients to the fact that there was no organized EDM program in their facility. Figure 2 shows frequency of client teaching by participants and Table 2 shows participants opinion of factors that can influence client teaching.

**Table 2:** Factors that can influence client teaching as indicated by participants

**Factors**	**Frequency**	**%**
Knowledge of breast cancer	70	60.9
Professional experience	88	76.5
Personal experience with breast cancer	59	51.3
Had a relation that had breast cancer	88	76.5
Concern about cost of management of advanced breast cancer	62	53.9
Work place policy/ norm in my work place	43	37.4
Response from client in the past	41	35.7
Desire for conservative management of breast cancer	56	48.7

Multiple linear regression of practice scores for BSE on knowledge of breast cancer, knowledge of EDM and age showed that for every unit increase in knowledge of breast cancer score, practice of BSE score significantly increased by about 0.45 units (95% CI = 0.06 to 0.85). There was also significant relationship between knowledge of EDM score and practice score of BSE, However there was no significant relationship between age of participant and their practice scores. Table 3 shows the details.

**Table 3:** **Multiple regression of practice scores for BSE on knowledge of breast cancer score**, **knowledge of EDM score and age**

**Variable**	**Regression coefficient (Beta)**	**95% CI for Beta**	**p-value**
Knowledge of breast cancer score	0.45	0.06 to 0.85	0.024
Knowledge of EDM score	0.46	0.15 to 0.76	0.004
Age	0.007	- 0.59 to 0.73	0.836

The logistic regression of client teaching on four variables showed that for every increase in knowledge of breast cancer the odds of client teaching significantly increased by 7.5% (95% CI = 1.03 - 1.13). There were also significant associations between knowledge of EDM, practice of BSE and client teaching. The odds of client teaching increased with higher BSE practice scores but decreased with knowledge of EDM, Table 4 shows the details.

**Table 4:** **Logistic Regression of client Teaching on Knowledge of breast Cancer score**, **Knowledge of EDM score**, **Practice Score of EDM and Professional qualification**

**Variables**	**Odd ratio**	**95% CI OR**	**P valve**
Knowledge score of Breast cancer	1.075	1.027 - 1.125	0.002
Knowledge score of EDM	0.962	0.930 - 0.995	0.025
Practice score of BSE	1.048	1.024 -1.072	0.001
Professional groups RN/RM vs. RN/RM/PH/CHO	1.34	0.487 – 3.687	0.571

## Discussion

The findings of this study show that participants had high knowledge score on various aspects of breast cancer. This is not the usual pattern among health care workers particularly nurses in developing countries. A previous study [13] reported poor knowledge of breast cancer risk factor among nurses in Karachi, Pakistan. Another study in Morocco [19] reported that only 43% of the nurses in their study had good knowledge. Similarly a study among teachers in Saudi Arabia reported limited knowledge of breast cancer [20].

In Nigeria various studies among nurses have also depicted low level of knowledge. A study among female health care workers reported that only 9.8% of nurses in their study had good knowledge of risk factors of breast cancer [19]. Another study [21] among female health care workers reported that although 14% of the whole group had excellent knowledge only two nurses, (1%) of the study population had excellent knowledge while 43% had poor knowledge. Furthermore another study [17] reported that “breast cancer and breast self-examination practices among nursing students were relatively high, 97.3%”.

This current study however observed issues of concern as 40% of PHC nurses in this study considered pain as an early warning sign of breast cancer and only 20% correctly identified that a painless lump in the breast may be an early warning sign of breast cancer. In response to an unstructured question there were many wrong responses for EDM such as “diagnostic radiology”. There is a consensus in the literature that nurses play a very vital role in ensuring cancer information dissemination, screening and care as they have more frequent interaction with clients and their relatives [14]. There is need that continuing education programmes for nurses on breast cancer and its early detection measures should examine critical areas to ensure that vital and correct information are communicated to clients. Such programmes should be consistent and structured to ensure their effectiveness.

Majority of participants in this study considered early detection and prompt treatment as an advantage of regular BSE practice while a little more than half considered developing breast cancer as the danger of irregular or non practice of BSE practice. These views may not be shared among nurses in developed countries where organized National screening program made more efficient EDMs available. Screening mammography can detect lumps before they are palpable and if followed up by immediate and adequate treatment it reduces mortality ranging from 12 to 70%, [6]. The efficacy of BSE as a screening method for breast cancer is however not established in the literature.

In previous studies in developing countries BSE is often the most acknowledged EDM as well as the most practiced. In this study BSE was the most acknowledged and most practiced of the EDMs. While less than half of the participants in this study claimed to practice BSE only about half of these performed it monthly in the past 12months. In a study among Jordanian nurses, low mean scores of EDM of breast cancer was reported but almost 90% practiced BSE. However only 18% practiced it monthly [22]. Mammography is the least acknowledged and least utilized EDM. The same pattern has been reported among female health care workers in Lagos and Benin City in Nigeria [18,21] and among female teachers in Saudi Arabia [20].

Many studies have reported nurses’ and health care workers’ knowledge of breast cancer and practice of its EDM. However there is dearth of information on nurses’ and health care workers’ professional role to clients in relation to breast cancer and it’s EDM. This study explored nurses’ conduct of CBE and Formal Planned Client Teaching (FPCT) of EDM. Findings as depicted in Figure 2 indicated that very few of the participants engaged in FPCT and “how to conduct BSE” was the most taught. The participants in this study demonstrated high level of knowledge but same was not transferred to their clients. Very few of the nurses conducted CBE but most of those who did were confident of their skill. When they discovered lumps appropriate steps were taken in referring the clients to doctors. If this practice is encouraged among health care workers especially nurses it would have both direct and indirect positive implications on early detection of breast cancer. It has been suggested that in the developing countries training should be extended to heath care workers [7] and not just be limited to the general public. Hence there is need to drive continuing education programmes on various aspects of breast cancer among Primary Health Care nurses whose focus of care is disease prevention and health promotion.

Socio-demographic characteristics of age, educational level and type of nursing school as well as professional qualification were not significantly associated with knowledge, practice or client teaching of breast cancer and EDM practices. Similarly a study among rural women [23] reported that in a group of rural women in Turkey there was no significant association between age groups and BSE practice. Furthermore, the authors reported that other studies have not found significant association between socio-demographic characteristics and BSE practice. However in a Singapore study [24] socio -demographic variables were not also significantly associated with BSE practice, but clinical experience with caring for a client with breast cancer was significantly associated with knowledge and practice.

In conformity with previous studies in Morocco [19] and Turkey [23], knowledge of breast cancer was a significant constant predictor of practice of EDM. In this study practice of BSE was also a significant constant predictor of client teaching, it is imperative therefore to integrate continuing education programs for nurses to ensure practice and client teaching. A national screening program is the ultimate. When this is put in place, nurses will be vital to its successful implementation. Nurses will have to utilize culture based strategies to ensure utilization as availability does not always ensure utilization because there are many factors that may hinder utilization as demonstrated in a previous study [6].

## Conclusions

In conclusion the findings of this study indicated good knowledge of breast cancer and its’ EDM. However the participants did not consider it appropriate to transfer knowledge to their clients. This may explain the report of poor knowledge and practice as well as perceptions by women from the same environment in previous studies [15,25]. The findings of the previous study motivated the current study. The need for nurses to understand their role in public education and client teaching need to be reinforced especially in an environment where there is no organized breast cancer screening. Nurses may organize formal planned client teaching and conduct CBE in their Primary Health Care units in order to assist them with self care practices and to help them maximize self-care abilities [26].

## Competing interest

I have no competing interest.

## Authors’ information

Dr. OAO is a Senior lecturer at the Department of Nursing, Faculty of Clinical Sciences, College of Medicine, University of Ibadan. She is a Community Health Nursing Specialist with research interest in women’s health and special interest in early detection and screening in women’s cancer.

## Pre-publication history

The pre-publication history for this paper can be accessed here:

http://www.biomedcentral.com/1472-6955/11/22/prepub
